# Backstabbing P-gp: Side-Chain Cleaved Ecdysteroid 2,3-Dioxolanes Hyper-Sensitize MDR Cancer Cells to Doxorubicin without Efflux Inhibition

**DOI:** 10.3390/molecules22020199

**Published:** 2017-01-25

**Authors:** Attila Hunyadi, József Csábi, Ana Martins, Joseph Molnár, Attila Balázs, Gábor Tóth

**Affiliations:** 1 Institute of Pharmacognosy, University of Szeged, 6720 Szeged, Hungary; csjoco88@gmail.com; 2 Interdisciplinary Centre for Natural Products, University of Szeged, Eötvös str. 6, 6720 Szeged, Hungary; 3 Department of Medical Microbiology and Immunobiology, University of Szeged, Dóm sq. 9, 6720 Szeged, Hungary; anasfmartins@gmail.com (A.M.); molnar.jozsef@med.u-szeged.hu (J.M.); 4 NMR Group, Department of Inorganic and Analytical Chemistry, Budapest University of Technology and Economics, Szt. Gellért Sq. 4, H-1111 Budapest, Hungary; balazs_attila_gyorgy@ymail.com (A.B.); drtothgabor@t-online.hu (G.T.)

**Keywords:** ecdysteroid metabolite, poststerone acetonide, ABCB1 efflux transporter, cancer, multi-drug resistance, chemo-sensitization, combination therapy

## Abstract

P-glycoprotein (P-gp, ABCB1) over-expression, causing a multi-drug resistant (MDR) phenotype, is a major problem in cancer chemotherapy that urgently requires novel approaches. Our previous studies showed certain ecdysteroid derivatives as promising chemo-sensitizers against MDR and non-MDR cancer cell lines while also exerting mild to moderate inhibition of P-gp function. Here we report the preparation of a set of substituted 2,3-dioxolane derivatives of poststerone, a known in vivo metabolite of 20-hydroxyecdysone (20E). In contrast with previously studied ecdysteroid dioxolanes, the majority of the new compounds did not inhibit the efflux function of P-gp. Nevertheless, a strong, dose dependent sensitization to doxorubicin was observed on a P-gp transfected cancer cell line and on its susceptible counterpart. We also observed that the MDR cell line was more sensitive to the compounds’ effect than the non-MDR. Our results showed for the first time that the chemo-sensitizing activity of ecdysteroids can be fully independent of functional efflux pump inhibition, and suggest these compounds as favorable leads against MDR cancer.

## 1. Introduction

Ecdysteroids have a broad range of bioactivities in mammals, as extensively reviewed elsewhere [1,2,3]. In addition to these, it has recently been revealed that less polar derivatives of these compounds can exert a potent chemo-sensitizing activity in various multi-drug resistant [4,5,6,7] as well as drug susceptible [6,7,8] cancer cell lines. In particular, ecdysteroid dioxolanes such as 20-hydroxyecdysone 2,3;20,22-diacetonide were found by our group to effectively potentiate the in vitro antitumor activity of several chemotherapeutic agents including doxorubicin, paclitaxel, and vincristine, which was, however, not the case for cisplatin [6]. The strong synergistic action of certain ecdysteroids with one or more of the above-mentioned chemotherapeutics was confirmed on a broad range of cancer cell lines of various origin, including human breast (MCF-7 and its sub-cell line MCF-7_Dox_ adapted to doxorubicin), prostate (PC3, LNCaP), epidermal (KB-3-1 and its sub-cell line KB-C-1 adapted to colchicine), and neuroblastoma (SH-SY5Y), as well as two murine lymphoma cell lines (L5178 and its sub-cell line L5178_MDR_ transfected to express the human ABCB1 transporter, commonly referred to as P-glycoprotein or P-gp) [4,5,6,7,8].

Over the last few years that have passed since the initial discovery of the chemo-sensitizing activity of these compounds [4], our research group has been pursuing related structure-activity relationships. In this endeavor, several important observations were made including the most recently reported effect of fluoride substitution on the activity [7]. In particular, the presence of apolar groups at positions 2 and 3 was found of outmost importance for the chemo-sensitizing potential, while such a substituent at the 20,22-diol appears to be of much less or if any significance [5]. Considering that the most abundant natural ecdysteroid, 20-hydroxyecdysone (20E) is known to undergo a side-chain cleavage between C-20 and C-22 during its in vivo metabolism to yield poststerone (**1**) [9], it seems reasonable to speculate that similar side-chain cleaved metabolites can also be formed from other ecdysteroids with a non-substituted diol at this position. Considering that such an in vivo metabolic alteration can apply to potential antitumor ecdysteroid derivatives containing apolar substituents on the 2,3-diol, the aim of the present study was to prepare such derivatives from poststerone (**1**) and to investigate their bioactivity as potential chemo-sensitizing agents on the L5178 cell line and on its P-gp transfected counterpart, L5178_MDR_.

## 2. Results and Discussion

### 2.1. Chemistry

**Figure 1:**
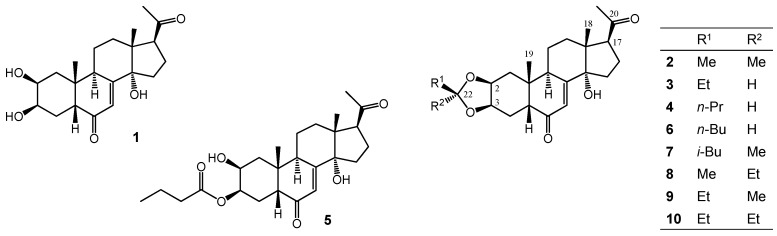
Chemical structures of compounds **1**–**10**.

Oxidative side-chain cleavage of 20E was achieved by using a common hypervalent iodine reagent (PIFA), and the subsequent purification by centrifugal partition chromatography (CPC) led to a relatively good, 57.8% isolated yield of poststerone (**1**). Following this, our aim was to prepare various substituted dioxolane rings fused to the ecdysteroid A-ring by utilizing different aldehydes or ketones reacting with the 2,3-diol similarly to our previously applied strategy [5]. Structure elucidation by NMR, performed as previously published in detail [10], revealed the chemical structures presented in Figure 1.

To facilitate the comparison of NMR data of products and the parental **1**, we measured and assigned the NMR signals of poststerone also in MeOH-*d*_4_, in which solvent they were not available before [11]. The corresponding ^1^H- and ^13^C-NMR shifts are compiled in Table 1 and Table 2, respectively.

**Figure 2:**
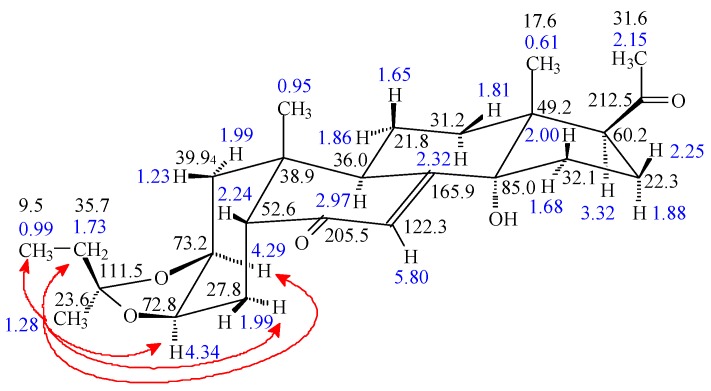
Stereostructure and one-dimensional selective Rotating frame Overhauser Enhancement Spectroscopy (ROESY) responses (irradiated at R^2^: *δ* = 1.28 ppm) of compound **8**. Blue numbers refer to ^1^H chemical shifts; black numbers give the ^13^C *δ* values.

A comparison of the *δ* H-2 and *δ* H-3 (3.86 and 3.97 ppm), moreover the corresponding *δ* C-2 and *δ* C-3 (68.7 and 68.5 ppm) values of the parental **1**, with the corresponding ^1^H and ^13^C chemical shifts obtained for the dioxolane derivatives (4.22–4.29 and 4.13–4.34 ppm) and (72.8–73.6 and 72.6–75.0 ppm) shows a pronounced deshielding. With the exception of the low-yield 3-acyl compound **5**, an unexpected by-product of compound **4** likely formed through the oxidation of an intermediate, the targeted dioxolane derivatives were obtained. Similar to our previous observation with analogous derivatives of 20E [5,10], whenever an asymmetrically substituted dioxolane ring was formed, the C-22 atom of the 2,3-dioxolane ring turns into a new stereogenic center. Steric effects caused the larger substituent on C-22 to be situated preferentially in the β-position [5,10] except for the present case of compounds **8** and **9** where the two possible 22-epimers were obtained in nearly identical yields. Their differentiation was then elucidated by one-dimensional Rotating frame Overhauser Enhancement (ROE) experiments, because this experiment in our case was much more effective than the analogous selective Nuclear Overhauser Enhancement (NOE) experiment. As shown in Figure 2, selective irradiation on the CH_3_ signal (1.28 ppm) of compound **8** resulted in strong ROESY responses on H-2 and H-3 signals, therefore their *cis* α positions as well as the *R* configuration of C-22 was unequivocally established.

### 2.2. Bioactivity

The poststerone derivatives **2**–**10** represented an ideal set of compounds to investigate the effect of a lacking side-chain on the chemo-sensitizing activity, and to study the relevant structure-activity relationships around C-2,3 in comparison with those concluded from our previous studies [4,5].

#### 2.2.1. Cytotoxic Activity and Functional Inhibition of P-Glycoprotein

Evaluation of the cytotoxicity of compounds **1**–**10** showed that, with the exception of compound **7**, the 50% inhibitory concentration (IC_50_) was higher than 75 μM, both on the L5178 and the L5178_MDR_ cell lines. Compound **7** had an IC_50_ equal to 59.6 ± 0.8 and 55.4 ± 4.9 μM against L5178 and L5178_MDR_, respectively.

The inhibitory effect on the efflux function was studied by measuring the compounds’ activity on the intracellular accumulation of rhodamine 123, a well-known P-gp substrate fluorescent dye, within the L5178_MDR_ cells. The efflux pump inhibitor tariquidar was used as positive control. Each compound was dissolved in DMSO whose final concentration (2%) was also evaluated for any effect on the retention of the fluorochrome, but no effect of the solvent was observed. The values were normalized to the fluorescence (FL) of rhodamine accumulated by L5178 and L5178_MDR_ cells such as: rhodamine 123 accumulation in the L5178 cells that do not overexpress the human P-gp, was considered as 100% P-gp inhibition while accumulation of Rhodamine 123 in L5178_MDR_ cells, that do overexpress the human P-gp, was considered as 0% inhibition. The obtained results are compiled in Table 3.

Compound **7** was the only one showing an effect in this bioassay, with approximately twice the activity of 20-hydroxyecdysone 2,3;20,22-diacetonide (20.91% inhibition at 20 μM, recalculated from data published in [4]); none of the other ecdysteroids exerted any significant inhibitory effect on the efflux function of P-gp. This indicates that lacking a side-chain significantly decreases this activity of apolar ecdysteroid derivatives, while a bulky 2,3-substituent, such as the methyl-isobutyl substituted dioxolane ring in compound **7**, can restore the P-gp inhibitory effect.

#### 2.2.2. Combination Studies with Doxorubicin

**Figure 3:**
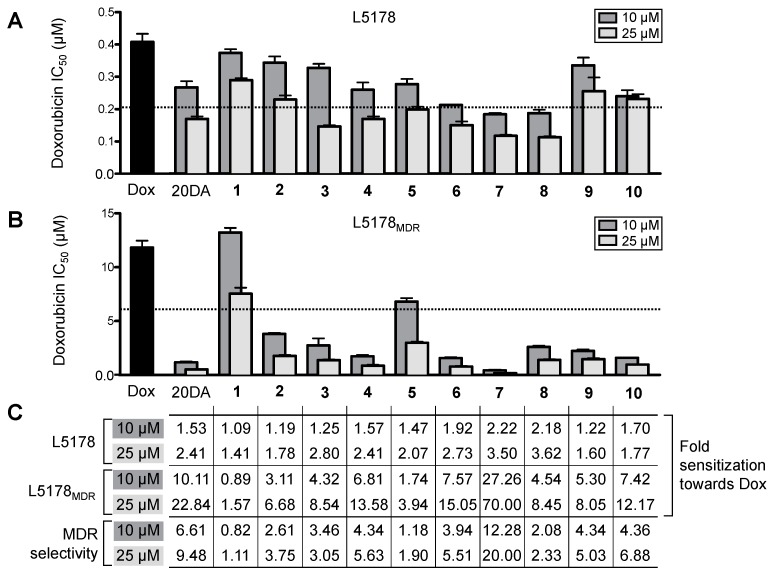
Chemo-sensitizing activity of compounds **1**–**10** on the two lymphoma cell lines. (**A**,**B**) Show the IC_50_ value of doxorubicin (Dox) alone or in combination with 10 or 25 μM of 20-hydroxyecdysone 2,3;20,22-diacetonide (20DA) or compounds **1**–**10**. The dashed line marks the threshold of two-fold sensitization. With the exception of 10 μM of compound **1** (either cell line), and **2**, **3**, and **9** (L5178), all IC_50_ values differ from that found for Dox alone at *p* < 0.01 by means of one-way ANOVA followed by Dunnett’s post-hoc test; IC_50_ values and levels of significance are provided as supporting information (Table S1); (**C**) Fold sensitization on both cell lines is presented, corresponding to how many times a certain concentration (10 or 25 μM) of the tested compound decreased the IC_50_ of Dox as compared to when it was used alone; MDR selectivity refers to the ratios of the IC_50_ values on the resistant and sensitive cell line: MDR selectivity = IC_50_(L5178_MDR_)/IC_50_(L5178).

Considering the negligible cytotoxic activity of each of the compounds, we evaluated the capacity of the compounds to potentiate the effect of doxorubicin. The effect of 10 or 25 μM of each compound was studied on the IC_50_ value of doxorubicin on both cell lines in order to allow evaluation of the effect of the missing side-chain on the chemo-sensitizing activity by comparing our new compounds’ activity with that of the non-substituted poststerone (**1**) as well as with our previous lead compound 20-hydroxyecdysone 2,3;20,22-diacetonide (20DA); results of this study are presented in Figure 3.

It is noteworthy, that all compounds showed a dose dependent chemo-sensitizing activity on both cell lines. With the exception of poststerone, **2**, **9,** and **10**, sensitization of the L5178 cells to doxorubicin was higher than 2-fold on the cells compared to when doxorubicin was applied alone, at least at the higher concentration (25 μM). On this cell line, the strongest activity was exerted by compounds **6**–**8**, each of which was significantly stronger than the diacetonide of 20E (20DA) when tested in a planned comparison by one-way ANOVA followed by Bonferroni’s post hoc test.

Concerning the transfected MDR cell line L5178_MDR_, compounds **2**–**6** and **8**–**10** showed remarkably strong chemo-sensitizing activity, even though they did not inhibit the efflux function of P-gp (in contrast with 20DA’s mild inhibition). As seen from Figure 3C, these compounds have up to 5–7 times stronger sensitizing effect on the MDR cells than on the parental cells, indicating that overexpression of P-gp by MDR cells induces a significant collateral sensitivity to the chemo-sensitizing activity of ecdysteroids. Exploiting collateral sensitivity—i.e., an evolutionary disadvantage connected to the MDR phenotype making such cells paradoxically hypersensitive to certain agents other than the ones they have become resistant to—appears to be a highly attractive strategy for overcoming MDR-related therapeutic difficulties [12]. A number of compounds have been identified that are able to exploit the fitness cost of resistance through various mechanisms [13]. In our case, further studies are necessary to clarify which mechanisms can be responsible for the observed MDR selective chemo-sensitizing activity, nevertheless, it appears clear that it can be completely separated from a functional efflux pump inhibition.

According to the structure-activity relationships (SARs), even though the acyl compound **5** was also active, the dioxolane derivatives were found favorable in this regard. Among these, a clear tendency was observed for gradually increasing the chemo-sensitizing activity on both cell lines when increasing the size of the β-substituent on the 2,3-dioxolane ring (in the order of compounds **2**, **3**, **4**, and **6**). Moreover, from the activities of compounds **8** and **9** it can also be seen that, in case of 22-epimers, the larger substituent is preferred to be in the 22 α-position (as in compound **8**) for a stronger sensitizing activity particularly on the non-MDR cell line. This extends our previous findings for the SAR observed at this position of side-chain bearing ecdysteroid dioxolanes [5] also to this series of compounds. Interestingly, however, the same difference between the bioactivites of compounds **8** and **9** was not observed on the L5178_MDR_ cells, which resulted in the higher MDR-selectivity of compound **9** as compared to **8**. In contrast with the other compounds investigated in this study, compound **7** could inhibit the efflux of rhodamine 123 by P-gp. Nevertheless, this compound showed a great sensitizing activity on both cell lines. It could completely reverse the resistance of the MDR cells at as low as 10 μM concentration, and at the higher dose (25 μM) it decreased the IC_50_ of doxorubicin from 0.41 and 11.8 μM (L5178 and L5178_MDR_, respectively) to 0.12 and 0.17 μM (L5178 and L5178_MDR_, respectively), highlighting its potential interest as a chemo-sensitizing agent.

## 3. Materials and Methods

### 3.1. General Information

The compound 20-hydroxyecdysone (20E) was purchased from Shaanxi KingsSci Biotechnology Co., Ltd. (Shanghai, China) at 90% purity and recrystallized from ethyl acetate/methanol (2:1, *v/v*) to reach a purity of 97.8% by means of HPLC–DAD. Reverse phase HPLC was performed on a system of two Jasco PU-2080 pumps connected to a Jasco MD-2010 Plus photodiode-array detector (Jasco Co., Tokyo, Japan). Normal phase HPLC was performed on a Waters 600 Pump connected to a Waters 2487 Dual λ Absorbance Detector (Waters Co., Milford, MA, USA). Mass spectra were recorded on an API 2000 triple quadrupole tandem mass spectrometer (AB SCIEX, Foster City, CA, USA) in positive mode with atmospheric pressure chemical ionization (APCI) ion source except for compounds **8**–**10**, which were measured with electron-spray ionization (ESI).

^1^H- (500.1 MHz) and ^13^C- (125.6 MHz) NMR spectra were recorded at room temperature on a Bruker Avance spectrometer and on Avance-III spectrometer (Bruker Biospin Co., Karlsruhe, Germany) equipped with a cryo probehead. Regarding the compounds, amounts of approximately 1–5 mg were dissolved in 0.1 mL of methanol-*d*_4_ and transferred to 2.5 mm Bruker MATCH NMR sample tube (Bruker). Chemical shifts are given on the *δ*-scale and are referenced to the solvent (MeOH-*d*_4_: *δ_C_* = 49.1 and *δ_H_* = 3.31 ppm). Pulse programs of all experiments (^1^H, ^13^C, DEPTQ, DEPT-135, sel-TOCSY, sel-ROE, edited gs-HSQC and gs-HMBC) were taken from the Bruker software library. The NMR signals of the product were assigned by comprehensive one- and two-dimensional NMR methods using widely accepted strategies [14,15]. Most ^1^H assignments were accomplished using general knowledge of chemical shift dispersion with the aid of the proton-proton coupling pattern (^1^H-NMR spectra).

### 3.2. Synthetic Procedure

Poststerone (**1**) was synthesized from 20E as follows: 2.0 g (4.17 mmol) of 20E was dissolved in 50.0 mL of methanol, and 2.7 g (1.5 equiv.) of [bis(trifluoroacetoxy)iodo]benzene (PIFA, Sigma-Aldrich, Budapest, Hungary) was added, and the reaction mixture was stirred at room temperature for 1 h. On completion, the mixture was quenched with 5% aqueous solution of NaHCO_3_ (Reanal Plc., Budapest, Hungary) and evaporated to dryness. The residue was dissolved in ethyl acetate, filtered through silica (Merck, Darmstadt, Germany) and dried in vacuo. The product was purified by centrifugal partition chromatography (Armen Spot CPC 250 mL, Armen Instrument, Saint Ave, France) in ascending mode with a solvent system of ethyl acetate/water/methanol (20:20:1, *v/v/v*), and 20 mL fractions were collected. Corresponding fractions were combined and dried to give poststerone (**1**), (871.6 mg, 57.8%).

Compounds **2**–**10** were synthesized from poststerone (**1**) according to the followings: 60 mg (0.166 mmol) of poststerone (**1**) was dissolved in 10 mL of methanol, then the reagent was added to the solution (**2**: acetone, 20 mL; **3**: propionaldehyde, 5 mL; **4** and **5**: butyraldehyde, 5 mL; **6**: valeraldehyde, 5 mL; **7**: methyl isobutyl ketone, 5 mL; **8** and **9**: methyl ethyl ketone, 5 mL; **10**: 3-pentanone, 5 mL). Catalytic amounts of *p*-toluenesulfonic acid (Sigma-Aldrich) were added, and the mixture was stirred at room temperature for two days in case of compound **2** and one week in case of compounds **3**–**10**. Then the reaction mixture was quenched with 5% aqueous solution of NaHCO_3_ and diluted with water. The mixture was concentrated by vacuum distillation until only water was present, and the aqueous solution was extracted three times with methylene chloride. The combined organic layers were dried with anhydrous Na_2_SO_4_ and evaporated to dryness. The products were isolated by rotational planar chromatography on silica gel with appropriate eluents composed of ethyl acetate and ethanol. Compounds **3** and **5**–**9** were further purified by semi-preparative HPLC using isocratic elution with aqueous methanol (63%, 70%, 68%, 75% for compounds **3**, **5**, **6**, and **7**, respectively, and 65% for compounds **8** and **9**) at a flow rate of 3.0 mL/min, by utilizing an Agilent Eclipse XDB-C8 (Agilent Technologies, Santa Clara, CA, USA) (9.4 mm × 250 mm, 5 μm) column. The yields were as follows: **2** (39.2 mg, 58.8%), **3** (16.7 mg, 25.1%), **4** (10.0 mg, 14.5%), **5** (7.3 mg, 10.2%), **6** (7.6 mg, 10.7%), **7** (1.5 mg, 2.0%), **8** (8.9 mg, 12.9%), **9** (7.0 mg, 10.2%), **10** (16.2 mg, 22.7%).

### 3.3. Compound Characterization Data

*Poststerone* (**1**): White crystals; m.p. 238–240 °C; for ^1^H- and ^13^C-NMR data, see Table 1 and Table 2, respectively; APCI-MS: 363 [M + H]^+^, 345 [M + H − H_2_O]^+^, 327, 309.

*22,22-Dimethyl-2,3-O-methylidene-poststerone* (**2**): White crystals; m.p. 185–186 °C; for ^1^H- and ^13^C-NMR data, see Table 1 and Table 2, respectively; APCI-MS: 403 [M + H]^+^, 385 [M + H − H_2_O]^+^, 359, 345, 327.

*22β-Ethyl-2,3-O-methylidene-poststerone* (**3**): White crystals; m.p. 82–83 °C; for ^1^H- and ^13^C-NMR data, see Table 1 and Table 2, respectively; APCI-MS: 403 [M + H]^+^, 385 [M + H − H_2_O]^+^, 359, 345, 327.

*22β-Propyl-2,3-O-methylidene-poststerone* (**4**): White crystals; m.p. 76–77 °C; for ^1^H- and ^13^C-NMR data, see Table 1 and Table 2, respectively; APCI-MS: 417 [M + H]^+^, 399 [M + H − H_2_O]^+^, 395, 377, 345, 327.

*Poststerone 3-butyrate* (**5**): White crystals; m.p. 95–97; for ^1^H- and ^13^C-NMR data, see Table 1 and Table 2, respectively; APCI-MS: 433 [M + H]^+^, 415 [M + H − H_2_O]^+^, 391, 377, 345, 327.

*22β-Butyl-2,3-O-methylidene-poststerone* (**6**): White crystals; m.p. 162–163 °C; for ^1^H- and ^13^C-NMR data, see Table 1 and Table 2, respectively; APCI-MS: 431 [M + H]^+^, 413 [M + H − H_2_O]^+^, 377, 359, 345, 327.

*22β-Isobutyl-22α-methyl-2,3-O-methylidene-poststerone* (**7**): White crystals; m.p. 195–197 °C; for ^1^H- and ^13^C-NMR data, see Table 1 and Table 2, respectively; APCI-MS: 445 [M + H]^+^, 427 [M + H − H_2_O]^+^, 423, 409, 391, 377, 359, 345, 327.

*22α-Ethyl-22β-methyl-2,3-O-methylidene-poststerone* (**8**): White crystals; m.p. 193–194 °C; for ^1^H- and ^13^C-NMR data, see Table 1 and Table 2, respectively; ESI-MS: 449 [M + CH_4_O]^+^, 417 [M + H]^+^.

*22β-Ethyl-22α-methyl-2,3-O-methylidene-poststerone* (**9**): White crystals; m.p. 88–89 °C; for ^1^H- and ^13^C-NMR data, see Table 1 and Table 2, respectively; ESI-MS: 449 [M + CH_4_O]^+^, 417 [M + H]^+^.

*22,22-Diethyl-2,3-O-methylidene-poststerone* (**10**): White crystals; m.p. 183–185 °C; for ^1^H- and ^13^C-NMR data, see Table 1 and Table 2, respectively; ESI-MS: 463 [M + CH_4_O]^+^, 431 [M + H]^+^.

### 3.4. Cytotoxicity Assay

Cytotoxicity activities on the L5178 and L5178_MDR_ cell lines were performed as described before [6]. Briefly, 10^4^ cells/well were incubated with serial dilutions of each compound (*n* = 3) in McCoy’s 5 A medium (Sigma-Aldrich) for 48 h at 37 °C, 5% CO_2_. Then, 3-(4,5-dimethylthiazol-2-yl)-2,5-diphenyltetrazolium bromide (MTT, Sigma) was added to each well at a final concentration of 0.5 mg/mL per well and after 4 h of incubation, 100 μL of sodium dodecyl sulfate (SDS) 10% (Sigma-Aldrich) in 0.01 M HCl was added to each well. Plates were further incubated overnight and the optical densities were read at 540 and 630 nm using an ELISA reader (Multiskan EX, Thermo Labsystem, Milford, MA, USA). Fifty percent inhibitory concentrations (IC_50_) were calculated using non-linear regression curve fitting of log (inhibitor) vs. response and variable slope with a least squares (ordinary) fit of GraphPad Prism 5 software (GraphPad Software Inc., San Diego, CA, USA). IC_50_ values were statistically evaluated by one-way ANOVA followed by Dunnett’s (with doxorubicin applied alone as control column) or Bonferroni’s post hoc test (with planned comparisons between the activities of compounds of particular interest).

### 3.5. Sensitization of L5178 and L5178_MDR_ Cells to Doxorubicin

Sensitization assay was performed in the same way as the cytotoxicity assay described above. However, in this assay doxorubicin was serially diluted in the 96 well plate and compounds **1**–**10** were added to each well at fix concentrations of 10 μM or 25 μM. In addition to the medium and cell control, wells that contained only cells and compound at the studied concentration were also included to assure that the concentrations used did not affect the cell growth by themselves.

### 3.6. Evaluation of P-glycoprotein Function through Rhodamine 123 Accumulation Assay

Inhibition of efflux function was evaluated using rhodamine 123, a fluorescent dye, whose retention inside the cells was evaluated by flow cytometry [4]. Briefly, 2 × 10^6^ cells/mL were treated with 2 and 20 μM of each compound. After 10 min incubation, rhodamine 123 (Sigma-Aldrich) was added to a final concentration of 5.2 μM and the samples were incubated at 37 °C in a water bath for 20 min. Samples were centrifuged (Heraeus Labofuge 400, Thermo Fisher Scientific, Waltham, MA, USA) (2000 rpm, 2 min) and washed twice with phosphate buffer saline (PBS, Sigma). The final samples were re-suspended in 0.5 mL PBS and its fluorescence measured with a Partec CyFlow flow cytometer (Partec, Münster, Germany). Tariquidar was kindly provided by Dr. Milica Pesic from the Institute for Biological Research Sinisa Stankovic, Belgrade, Serbia, and it was used at 50 nM as positive control.

## 4. Conclusions

Through the synthesis and bioactivity testing of a series of poststerone 2,3-dioxolanes, we have shown for the first time that structure-activity relationships connected to the chemo-sensitizing activity of ecdysteroids can in fact be separated from those describing their inhibitory effect on P-gp function. On the one hand, this strongly supports our previous assumption, namely that a mechanism(s) other than a functional P-gp inhibition must primarily be responsible for the chemo-sensitizing activity of less-polar ecdysteroid derivatives, and P-gp inhibition, if any, is rather a side-effect to this. On the other hand, side-chain cleaved ecdysteroid 2,3-dioxolanes, originated from the in vivo ecdysteroid metabolite poststerone, are hereby suggested as promising, non-P-gp inhibitor MDR selective adjuvant agents for further development.

## Figures and Tables

**Table 1:** ^1^H-NMR chemical shifts of compounds **1**–**10**, in ppm, in MeOH-*d*_4_.

Atom No.	1	2 ^a^	3	4	5	6	7	8	9	10 ^a^
1 β	1.44	1.24	1.18	1.17	1.44	1.18	1.23	1.26	1.23	1.25
α	1.80		2.01	2.00	1.94	2.01	1.98	2.00	1.99	
2	3.86	4.28	4.23	4.23	4.00	4.22	4.27	4.29	4.29	4.29
3	3.97	4.31	4.13	4.13	5.17	4.13	4.31	4.27	4.34	4.32
4 β	1.74		2.02	2.02	1.78	2.02	1.98	1.98	1.99	
α	1.74		2.02	2.02	1.78	2.02	1.98	1.98	1.99	
5	2.39		2.25	2.25	2.22	2.25	2.23	2.25	2.24	
7	5.82	5.80	5.81	5.80	5.83	5.80	5.80	5.80	5.80	5.80
9	3.19	2.98	2.99	2.99	3.21	2.99	2.97	2.97	2.97	2.96
11 β	1.67		1.67	1.67	1.69	1.66	1.66	1.66	1.65	
α	1.89		1.88	1.88	1.89	1.87	1.87	1.86	1.86	
12 β	1.82		1.81	1.81	1.83	1.81	1.80	1.81	1.81	
α	2.33	2.32	2.32	2.32	2.35	2.32	2.32	2.32	2.32	2.32
15 β	2.00		2.00	2.02	2.01	2.01	1.99	2.00	2.00	
α	1.70		1.69	1.69	1.69	1.69	1.69	1.68	1.68	
16 β	2.23		2.25	2.26	2.26	2.25	2.24	2.25	2.25	
α	1.88		1.88	1.90	1.89	1.89	1.88	1.89	1.88	
17	3.33	3.32	3.32	3.32	3.33	3.33	3.33	3.34	3.32	3.32
18	0.62	0.62	0.62	0.62	0.63	0.62	0.61	0.62	0.61	0.61
19	0.96	0.96	0.96	0.96	0.99	0.96	0.96	0.97	0.95	0.96
21	2.16	2.15	2.15	2.15	2.16	2.15	2.15	2.15	2.15	2.15
R^1^	-	1.47 -	0.98 1.68 -	0.97 1.46 1.65 -	0.99 1.69 2.40 -	0.93 1.39 1.40 1.67	0.98 0.98 1.83 1.63	1.42 -	0.90 1.73 -	0.95
R^2^	-	1.32 -	4.90 -	4.95 -	-	4.94 -	1.30 -	0.92 1.62	1.28 -	0.88

^a^ Only the characteristic ^1^H chemical shifts were assigned for compounds **2** and **10**, considering that these compounds contain identical R^1^ and R^2^ groups, hence diastereomer pairs at C-22 were not distinguishable.

**Table 2:** ^13^C-NMR chemical shifts of compounds **1**–**10**, in ppm, in MeOH-*d_4_*.

Atom No.	1	2	3	4	5	6	7	8	9	10
1	37.4	38.8	39.5	39.5	38.5	39.6	39.0	39.0	38.94	39.1
2	68.7	73.6	72.9	72.8	67.2	72.8	73.2	73.2	73.2	72.8
3	68.5	73.2	75.0	75.0	71.6	75.0	72.6	73.6	72.8	73.2
4	32.9	27.8	27.8	27.8	30.5	27.8	27.8	27.8	27.8	27.8
5	51.9	52.6	52.6	52.6	52.8	52.6	52.8	52.6	52.6	52.6
6	206.3	205.5	205.3	205.3	205.2	205.3	205.5	205.4	205.5	205.4
7	122.6	122.3	122.4	122.3	122.5	122.3	122.3	122.3	122.3	122.3
8	166.6	165.9	166.0	166.0	166.8	166.0	166.0	165.9	165.9	165.9
9	35.2	35.9	36.3	36.3	35.4	36.3	36.1	36.0	36.0	36.0
10	39.3	39.0	38.8	38.7	39.3	38.8	38.9	38.9	38.9	38.9
11	21.7	21.8	21.9	21.9	21.7	21.9	21.8	21.8	21.8	21.8
12	31.1	31.2	31.2	31.2	31.2	31.2	31.2	31.2	31.2	31.2
13	48.9	49.1	49.2	49.2	48.8	49.1	49.4	49.2	49.2	49.0
14	85.1	85.0	85.0	85.0	85.1	85.0	85.1	85.0	85.0	85.0
15	32.2	32.1	32.1	32.1	32.2	32.1	32.1	32.1	32.1	32.1
16	22.3	22.3	22.3	22.3	22.3	22.3	22.3	22.3	22.3	22.3
17	60.2	60.2	60.2	60.2	60.2	60.2	60.2	60.2	60.2	60.2
18	17.6	17.6	17.6	17.6	17.6	17.6	17.6	17.6	17.6	17.6
19	24.5	24.1	24.1	24.1	24.5	24.1	24.2	24.1	24.1	24.2
20	212.6	212.6	212.5	212.5	212.5	212.5	212.5	212.5	212.5	212.5
21	31.6	31.6	31.6	31.6	31.6	31.6	31.6	31.6	31.6	31.6
22	-	109.6	106.7	105.7	175.1	105.9	111.4	111.7	111.5	113.5
R^1^	-	28.9 -	8.80 29.5 -	14.5 18.8 38.7 -	14.1 19.6 37.3 -	14.4 23.8 27.7 36.7	24.9 24.9 26.1 51.8	25.7 -	9.5 35.7 -	9.07 31.7 -
R^2^	-	26.7 -	-	-	-	-	24.4 -	9.40 33.4	23.6 -	9.11 29.6

**Table 3:** Functional inhibition of P-gp by compounds **1**–**10**. ^a^

Compound	Inhibition (%)		Compound	Inhibition (%)	
	2 μM	20 μM		2 μM	20 μM
**1**	0.31	0.43	**6**	−0.06	2.32
**2**	0.08	0.64	**7**	3.08	56.36
**3**	0.24	0.29	**8**	−0.19	0.19
**4**	0.03	3.85	**9**	−0.32	−0.16
**5**	−0.19	0.03	**10**	−0.30	0.11

^a^ Positive control: 50 nM of tariquidar (109.59% inhibition), negative control: 2% DMSO (−0.42% inhibition).

## Supplementary Materials

Supplementary materials can be accessed at: http://www.mdpi.com/1420-3049/22/2/199/s1. Figure S1: ^1^H-NMR spectrum of compound **8**, Figure S2: DEPTQ spectrum of compound **8**, Figure S3: ^1^H-NMR spectrum of compound **9**, Figure S4: DEPTQ spectrum of compound **9**, Figure S5: Confirmation of the stereochemistry of compound **9** at C-22, Table S1: Cytotoxicity of doxorubicin alone and in combination with 10 or 25 μM of compounds **1**–**10** on the L5178 and L5178_MDR_ cell lines.
