# Supplementary material for: Backstabbing P-gp: Side-Chain Cleaved Ecdysteroid 2,3-Dioxolanes Hyper-Sensitize MDR Cancer Cells to Doxorubicin without Efflux Inhibition
Source: Molecules. 2017 Jan 25;22(2):199. doi: 10.3390/molecules22020199 (PMC6155823; doi:10.3390/molecules22020199)
Supplement: Supplementary file 1 [file molecules-22-00199-s001.pdf]

Supplementary Materials: Backstabbing P-gp: Side-Chain Cleaved Ecdysteroid 2,3-Dioxolanes Hyper-Sensitize MDR Cancer Cells to Doxorubicin without Efflux Inhibition

Attila Hunyadi, József Csábi, Ana Martins, Joseph Molnár, Attila Balázs and Gábor Tóth

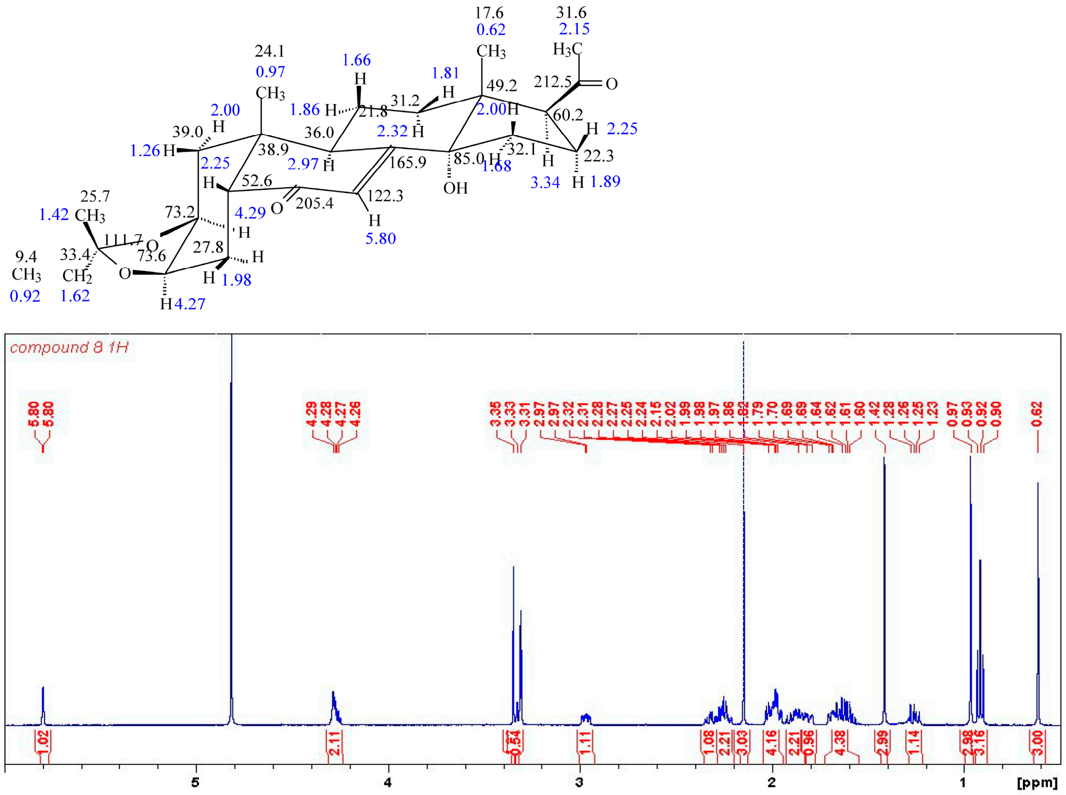

Figure S1. ^{1}H-NMR spectrum of compound **8**.

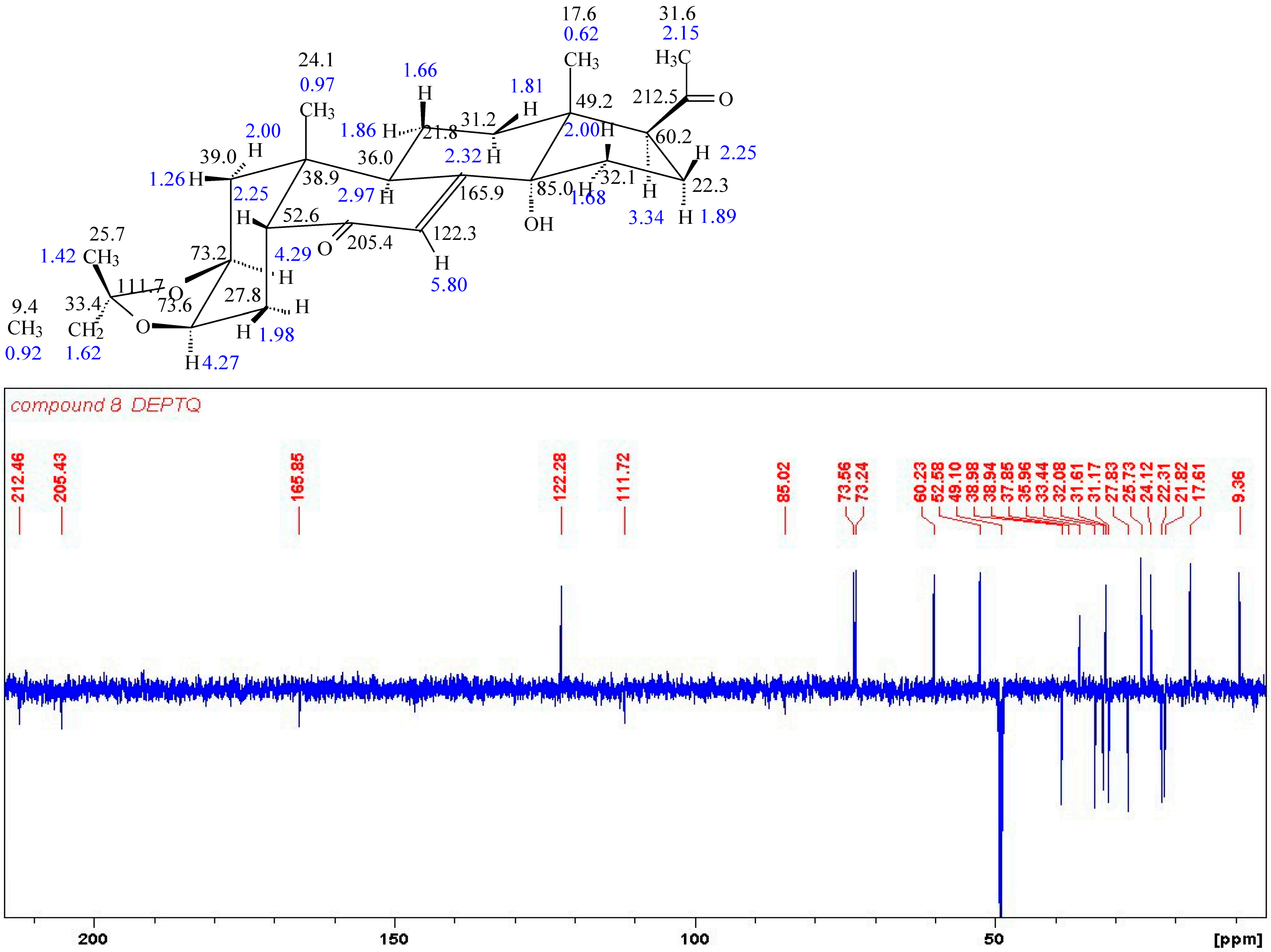

Figure S2. DEPTQ spectrum of compound **8**.

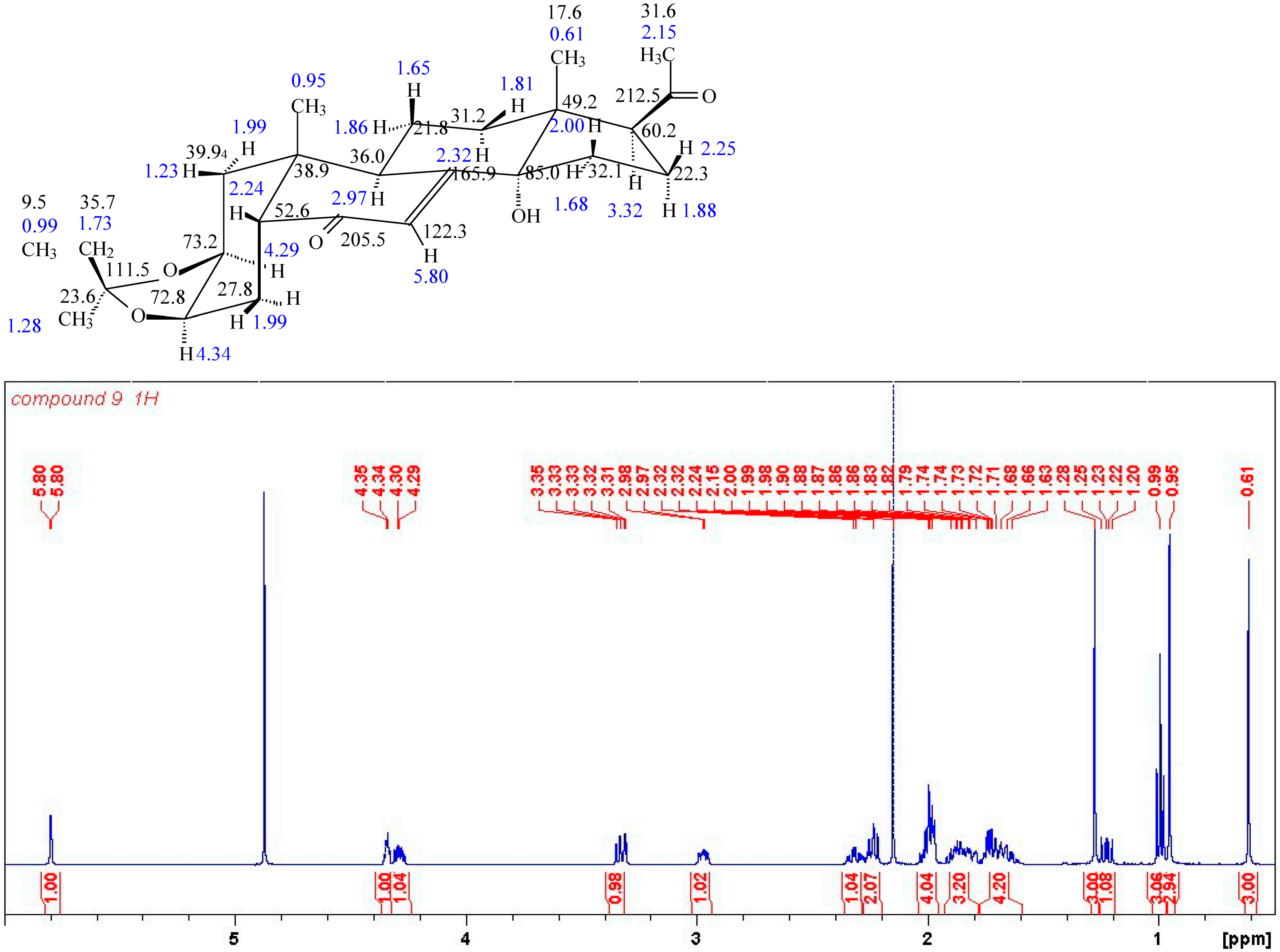

Figure S3. ^{1}H-NMR spectrum of compound **9**.

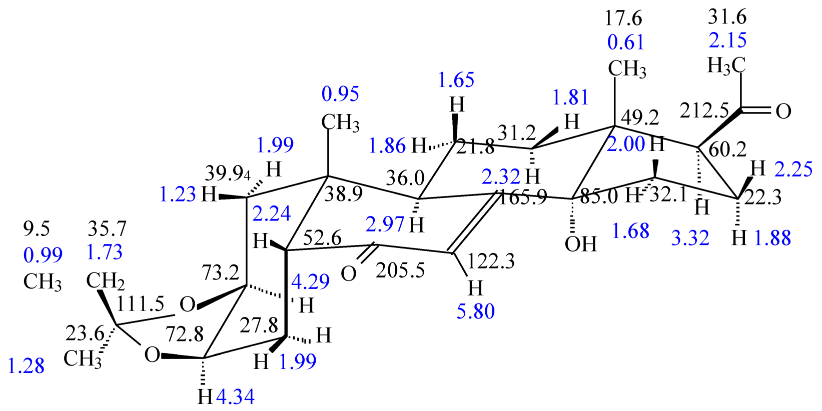

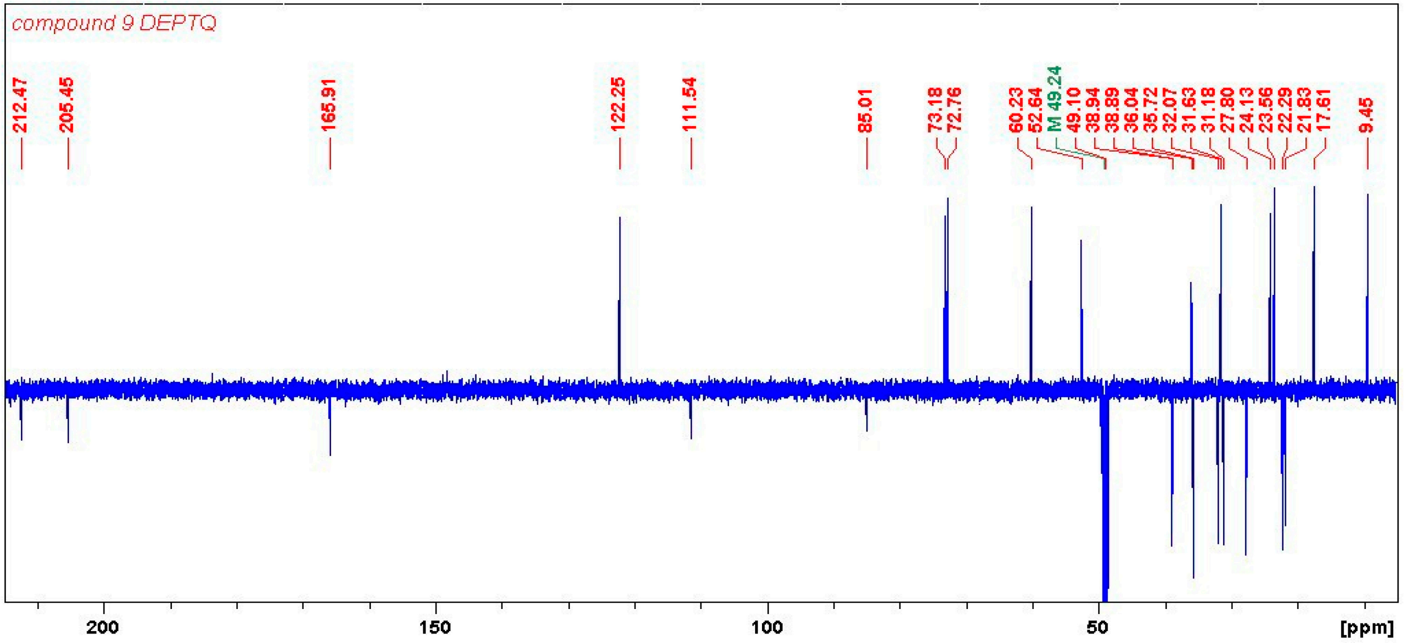

Figure S4. DEPTQ spectrum of compound **9**.

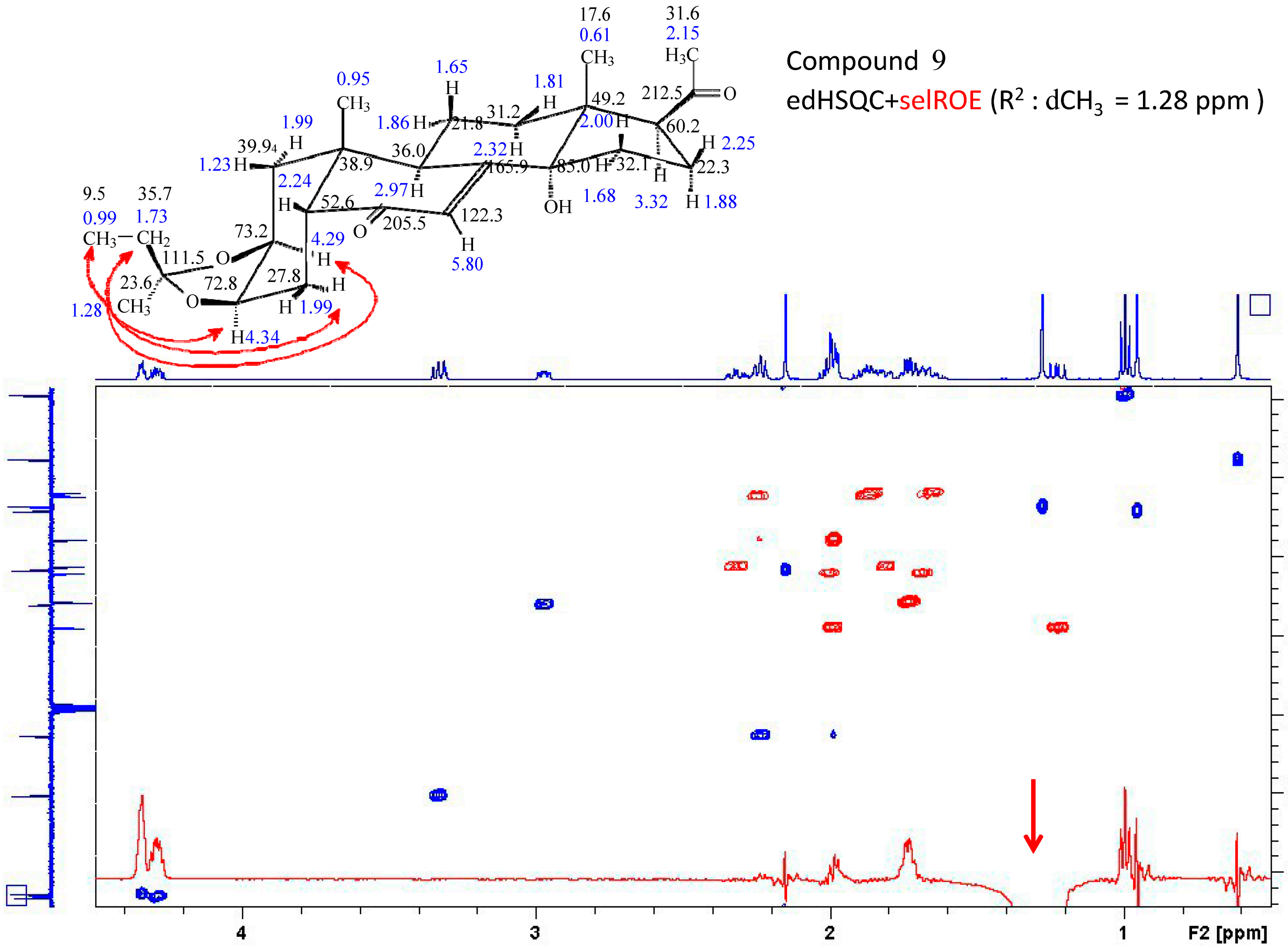

Figure S5. Confirmation of the stereochemistry of compound **9** at C-22.

Table S1. Cytotoxicity of doxorubicin alone and in combination with 10 or 25 μM of compounds **1–10** on the L5178 and L5178MDR cell lines.

			IC₅₀ of Doxorubicin (μM)										
**Cell Line**	**Doxorubicin Alone**	**+ Ecdysteroid**	**20DA**	**1**	**2**	**3**	**4**	**5**	**6**	**7**	**8**	**9**	**10**
L5178	0.41 ± 0.02	10 μM	0.27 ± 0.03 **	0.37 ± 0.02	0.34 ± 0.03	0.33 ± 0.02	0.26 ± 0.02 **	0.28 ± 0.02**	0.21 ± 0.003**	0.18 ± 0.005 **	0.19 ± 0.02 **	0.34 ± 0.04	0.24 ± 0.03 **
		25 μM	0.17 ± 0.01 **	0.29 ± 0.01 *	0.23 ± 0.02 **	0.15 ± 0.006 **	0.17 ± 0.01 **	0.20 ± 0.02 **	0.15 ± 0.02 **	0.12 ± 0.003 **	0.11 ± 0.003 **	0.26 ± 0.07 **	0.23 ± 0.03 **
L5178MDR	11.8 ± 0.64	10 μM	1.17 ± 0.08 **	13.22 ± 0.44	3.80 ± 0.10 **	2.74 ± 0.66 **	1.74 ± 0.11 **	6.81 ± 0.33 **	1.56 ± 0.14 **	0.43 ± 0.02 **	2.60 ± 0.11 **	2.23 ± 0.14 **	1.60 ± 0.02 **
		25 μM	0.52 ± 0.05 **	7.55 ± 0.56 **	1.77 ± 0.08 **	1.39 ± 0.06 **	0.87 ± 0.08 **	3.00 ± 0.09 **	0.79 ± 0.05 **	0.17 ± 0.005 **	1.34 ± 0.08 **	1.47 ± 0.08 **	0.97 ± 0.03 **

Results are given in μM as mean ± SEM; *: p <0.01, **: p <0.001 by means of one-way ANOVA followed by Dunnet's post hoc test as compared to the IC_{50} value of doxorubicin alone, n = 3. Values representing higher than two-fold sensitization to doxorubicin are highlighted in blue, those with higher than five-fold, in red.
